# A26 DECONSTRUCTING DISTRESS: STAKEHOLDER ENGAGEMENT FOR EVIDENCE-BASED, PATIENT-CENTERED INTERVENTIONS FOR THE MANAGEMENT OF IBD-ASSOCIATED PSYCHOLOGICAL DISTRESS

**DOI:** 10.1093/jcag/gwab049.025

**Published:** 2022-02-21

**Authors:** C Heisler, N Rohatinsky, M Stewart, M Vallis, T Shepherd, L Wozney, C Cassidy, B Currie, K Phalen-Kelly, J Robar, L E Targownik, T Huard, E Neil, J Jones

**Affiliations:** 1 Gastroenterology, Research Services, QEII Health Sciences Centre, Halifax, NS, Canada; 2 Medicine, Dalhousie University, Halifax, NS, Canada; 3 QEII Health Sciences Centre, Halifax, NS, Canada; 4 University of Saskatchewan, Saskatoon, SK, Canada; 5 Nova Scotia Health Authority, Halifax, NS, Canada; 6 University of Toronto, Toronto, ON, Canada

## Abstract

**Background:**

The growing prevalence of Inflammatory Bowel Disease (IBD) along with increasing complexity of providing high-quality, patient-centered care within a resource-constrained healthcare environment presents a major challenge. IBD-related psychological distress (IBD-PD) is the emotional impact of IBD and is associated with mental health disorders, increased disease severity, and premature mortality. With estimates of nearly 90% of IBD patients experiencing PD, the inability to provide high-quality, person-centered care for IBD-PD that is proportionate to clinical need is a significant care gap in the Canadian healthcare system.

**Aims:**

To generate stakeholder-derived data to inform the design and development of stepped-intensity, cognitive behavioral therapy-based interventions for IBD-PD using evidence-based, patient–centered interventions and implementation strategies.

**Methods:**

Virtual semi-structured interviews were conducted from September to October 2021. The interview guide was developed iteratively by researchers, IBD care providers, and patient research partners and guided by the COM-B Model of Behaviour and the Theoretical Domains Framework. Questions assessed perceptions, experiences, barriers, and facilitators to accessing IBD-PD care. Adults diagnosed with IBD were recruited from academic centers across Canada. Interviews were co-facilitated by a researcher and patient research partner, audio recorded, and transcribed. Using thematic analysis, codes were generated to identify themes using an inductive approach.

**Results:**

As of October 2021, six interviews have been completed, with data collection ongoing. The mean participant age was 34.3 years (range 21–55 years) with 100% of respondents being female. The majority of participants worked full time (4/6, 67%) and all had completed at least high school. Diagnoses of Crohn’s Disease (3/6, 50%) and ulcerative colitis (3/6, 50%) were evenly distributed. Thematic analyses identified five major themes: 1) Lack of holistic care and acknowledgement of IBD-PD; 2) System-level and financial barriers to psychological support; 3) Lack of psychological support from providers with an understanding of IBD; 4) Preference for individualized virtual-based support; 5) Heavy reliance on informal support structures (caregivers) due to lack of access to formal psychological support.

**Conclusions:**

As part of human-centered design, stakeholder engagement is key to understanding behavioral, social, attitudinal, and environmental barriers and facilitators for accessing IBD-PD care. Interviews are ongoing and specific intervention functions will be defined and incorporated into patient-centered implementation strategies.

**Funding Agencies:**

None

